# Active immunization using exotoxin A confers protection against *Pseudomonas aeruginosa *infection in a mouse burn model

**DOI:** 10.1186/1471-2180-9-23

**Published:** 2009-02-01

**Authors:** Ali Manafi, Jamshid Kohanteb, Davood Mehrabani, Aziz Japoni, Masoud Amini, Mohsen Naghmachi, Ahmad Hosseinzadeh Zaghi, Nazanin Khalili

**Affiliations:** 1 Department of Plastic Surgery, School of Medicine, Iran University of Medical Sciences, Tehran, Iran; 2 Department of Medical Microbiology, School of Medicine, Shiraz University of Medical Sciences, Shiraz, Iran; 3 Gastroenterohepatology Research Center, Nemazee Hospital, Shiraz University of Medical Sciences, Shiraz, Iran; 4 Professor Alborzi Clinical Microbiology Research Center, Shiraz University of Medical Sciences, Shiraz, Iran; 5 Department of Surgery, Shiraz University of Medical Sciences, Shiraz, Iran; 6 Yasuj University of Medical Sciences, Yasuj, Iran; 7 School of Pharmacy, Shiraz University of Medical Sciences, Shiraz, Iran

## Abstract

**Background:**

*Pseudomonas aeruginosa *is an important cause of nosocomial infection and may lead to septicemia and death. We evaluated the immunogenicity of semi-purified exotoxin A from the bacterium in a mouse burn model.

**Methods:**

The toxoid was prepared from exotoxin A taken from toxigenic strains of *P. aeruginosa *(PA 103). 50 mice were immunized with the toxoid, burned with hot metal and infected with 1 × 10^8 ^CFU of toxigenic strains of *P. aeruginosa *(experimental group); 25 non-immunized mice were also burned and infected (control group). The mortality rate and presence of any exotoxin and *P. aeruginosa *in the sera, liver and spleen were determined.

**Results:**

In the experimental group, 2 mice died before the burns were administered and were excluded from the study. The remainder (48 mice) were challenged with a lethal dose of *P. aeruginosa *and followed for 70 days. 3 of these mice died. Neither *P. aeruginosa *nor exotoxin A was not detected in the liver, spleen or sera of the surviving mice. The protective efficacy of toxoid vaccination was therefore 93.8%. In the control group, all mice died from bacteremia and septicemia, most (80%) within 6 days, and *P. aeruginosa *and exotoxin A were isolated from sera, spleen and liver.

**Conclusion:**

Active immunization of mice using a semi-purified exotoxin A derived from *P. aeruginosa *was 93.8% effective at protecting mice from subsequent *P. aeruginosa *infections in a mouse burn model.

## Background

*Pseudomonas aeruginosa *is an opportunistic, non-fermentative, gram-negative rod which is an important cause of nosocomial infection leading to septicemia and death [1]. The mortality rate is higher than bacteremias caused by other gram-negative opportunistic pathogens. One of the most important features of the bacterium is its resistance to various antibacterial agents [2,3], and even newly developed antibiotics have failed to reduce the mortality rate associated with this organism [4].

There is increasing interest in bacterial virulence factors as a basis for effective vaccines and immunotherapies. Several extracellular products from *P. aeruginosa *such as exotoxin A, exoenzyme S, phospholipase and hemolysins have been studies as potential virulence factors [5]. The role of exotoxin A in the mortality of experimentally-infected animals has been demonstrated [6] and the LD50 of the exotoxin reported to be 60–80 ng/mouse [7]. Following a single injection of 80 ng of exotoxin A, necrosis, and cellular swelling were detected in liver within 48 h [7]. Hemorrhage in the lungs and necrosis in the kidneys were also reported [7,8]. In eukaryotic cells, when exotoxin A turns into an activated enzyme, transfer of an adenosine diphosphate ribose moiety from NAD led to inactivation of elongation factor 2 and inhibition of protein synthesis [7]. Furthermore, the pre-existence of a high titer of anti-exotoxin A antibody reportedly increased the survival rate in patients with *P. aeruginosa *bacteremia [9].

This study was performed to determine the immunogenicity of a toxoid produced from exotoxin A of *P. aeruginosa *in a mouse burn model.

## Methods

### Preparation of exotoxin A

A toxigenic strain of *P. aeruginosa *(PA 103) was used for exotoxin A preparation. Exotoxin A was partially purified according to the method described by Pollack et al. [10] and Homma et al. [11]. *P. aeruginosa *was inoculated into tryptic soy agar and incubated at 37°C for 24 h in ambient conditions. The growth product of the slant cultures was inoculated into 500 mL of Muller-Hinton broth and incubated at 37°C for another 24 h in ambient conditions. The bacterial suspension was centrifuged for 30 min at 2000 g and the supernatant containing exotoxin A was sterilized by the Millipore filtration method (0.45 μm) and concentrated 10× by polyethylene glycol (PEG) in a dialysis bag (30 mm diameter, Biogen, Mashhad, Iran). 200 mL of the concentrated supernatant was mixed with 200 mL of diethyl amino ethyl cellulose and stirred at 4°C. Exotoxin A was precipitated by the addition of 0.25 M of NaCl and 70% saturated ammonium sulfate. The precipitate was dissolved in 0.1 M of Tris hydrochloride buffer containing 0.5 M of NaCl and 0.02% of NaN3 (pH 8 at 4°C) and then applied into a column packed with Sephadex G75. The various fractions were collected and concentrated in dialysis bags (10 mm diameter, Biogen, Mashhad, Iran). Concentrated semi-purified exotoxin A was examined for presence of exotoxin A using the counter immunoelectrophoresis (CIEP) method. The protein content of exotoxin A was adjusted to 50 μg/mL by a spectrophotometer and used to immunize the mice.

### Animal selection

75 white out-bred mice were provided from the Laboratory Animal Research Center of the Shiraz University of Medical Sciences, housed in an ambient temperature of 21 ± 2°C and relative humidity of 65–70%, and given a balanced diet with free access to food and water. Animal selection, all experiments, subsequent care and the sacrifice procedure were all performed according to the guidelines and under the supervision of the Animal Care Committee of the Iran Veterinary Organization. The protocol for anesthesia, burn induction, post-burn care and sacrifice were identical for all animals. The animals were sacrificed under deep ether general anesthesia. All experiments were carried out under aseptic conditions. The study was approved by the Ethics Committee of the Shiraz University of Medical Sciences.

### Determination of LD50

To determine the LD50 of the exotoxin, 50 additional mice were divided into 10 equal groups. A series of dilutions, up to ten-fold, of 50 μg/mL of semi-purified exotoxin A were prepared in PBS (pH 7.2). Each of the 10 groups was assigned to one of the 10 dilutions, and 1 mL of solution was injected intraperitoneally in each animal. Therefore, the mice received between 0.0005 and 5 μg of exotoxin A. The mice were followed for 30 days. The LD50 was determined according to the Reed and Muench method [13] and calculated to be 0.5 μg.

### Preparation of toxoid

To prepare the toxoid, 5 mL of semi-purified exotoxin A was mixed with 10 mL of PBS, pH 7.2, containing 0.01 M sodium phosphate, 0.15 M sodium chloride and 4% formaldehyde, and incubated at 37°C for 4 days before being dialyzed against phosphate buffer for 48 h. The attenuated toxin was sterilized by Millipore filtration (0.45 μm).

### Mice immunization with toxoid

50 mice were assigned to the experimental group. 2 mice died before the burns were administered and were not enrolled in the study. The remaining 48 mice were immunized with the toxoid. Each mouse received weekly subcutaneous injections for 6 weeks. Each injection contained 100 μg of semi-purified toxoid in 2 mL of PBS. 1 week after the last injection, the animals were bled from the eye and the samples checked for the presence of antitoxin using CIEP. To determine more precisely the ranges of immunity in the vaccinated mice, the titer of anti-exotoxin A was measured by enzyme-linked immunosorbent assay (ELISA) as previously described [14].

### Rabbits hyperimmunization with toxoid

A group of 4 rabbits were immunized with the toxoid. Each rabbit received weekly subcutaneous injections for 6 weeks. Each injection contained 200 μg of semi-purified toxoid in 4 mL of PBS. 1 week after the last injection, the animals were bled from the ear. Sera were pooled and the presence of antitoxin against *P. aeruginosa *confirmed by CIEP. The sera were used as an antitoxin when necessary, to evaluate the presence of the toxin in the sera of the experimental and control mice.

### Counterimmunoelectrophoresis

CIEP was carried out for qualitative detection of toxin and antitoxin in the sera of the immunized mice [12]. This technique was applied on 13 × 18 cm glass slides which were covered by 1% melted agarose in acetate buffer (pH 7.6). 2 rows of wells with a diameter of 6 × 6 mm were punched in each glass slide and 0.4 mL of semi-purified exotoxin A or serum containing the exotoxin A (antigen) and 0.4 mL of immunized mice or rabbit serum (antibody) were placed in the anodal and cathodal wells, respectively. The slide was subjected to electrophoresis using an acetate buffer (pH 7.6 at 40 mA for 30 min). Production of a precipitation line between the two wells indicated the presence of antitoxin or toxin A in the sera. The Amidoblack staining method was used to reveal the precipitation lines more clearly.

### Determining the efficacy of the candidate vaccine

73 mice (48 immunized = experimental group, 25 non-immunized = control group) were anesthetized and burns (grade 3) were induced on the thigh using a 1 × 2 cm piece of hot metal, producing a burn of up to 10% of the total body surface and extending to all layers of skin but not involving the muscular tissue. After 24 h, 10^8 ^colony forming units (CFU) of toxigenic strains of *P. aeruginosa *(PA 103) were inoculated subcutaneously into the burned area.

Both groups were supervised in their cages for 70 days. Samples were obtained from the infected areas using sterile swabs and saline and checked for the presence of *P. aeruginosa *at different time intervals. Blood samples and the tissue samples of spleens and livers of dead mice were also examined for presence of *P. aeruginosa*. The presence of *P. aeruginosa *was determined as CFU/mL of the blood samples. The quantity of *P. aeruginosa *in the spleens and livers was measured as the number of CFU per 1 g of homogenized tissue. The survival rate in both groups was compared. The efficacy of vaccine was calculated as the percentage survival during the 70-day observation period following inoculation with toxogenic *P. aeruginosa *(PA 103).

### Confirmation of infection

Using sterile swabs and saline, samples were obtained from the infected burns. The swabs were cultured on blood and Muller-Hinton agar plates and incubated at 37°C under ambient conditions for 24 h. *P. aeruginosa *was diagnosed by colony morphology, a zone of hemolysis and oxidase, methyl red, Voges Proskauer, citrate and TSI tests [15].

## Results and discussion

Mice immunized with a semi-purified exotoxin A from *P. aeruginosa *(n = 48) and non-immunized mice (n = 25) received full-thickness burns to the skin of the thigh and were then challenged with 10^8 ^CFU of *P. aeruginosa *(a lethal dose). They were followed for 70 days. Antitoxin and exotoxin A were detected in the sera of the experimental group by CIEP. The antibody titer ranged from 1:16 to 1:512 in the immunized mice using ELISA (Table 1).

**Table 1:** Antitoxin titer of immunized mice using ELISA

Antitoxin titer	No. (%)
1:16	2 (4.5)
1:32	8 (17.8)
1:64	10 (22.2)
1:128	15 (33.3)
1:256	5 (11.1)
1:512	5 (11.1)

During the follow-up period, 3 mice (6.3%) in the experimental group died. All non-immunized mice developed septicemia and died within 3 weeks of inoculation with *P. aeruginosa*. In serial wound swabs (diluted in 1 ml of distilled water) from the immunized mice, 1.5 × 10^8 ^CFU/mL of *P. aeruginosa *were detected 1 day after wound inoculation and levels decreased to 0 over 2 weeks. In the non-immunized mice, the colony count increased for 6 days post-inoculation with *P. aeruginosa *and the majority of the mice (80%) died within this period. Table 2 shows the colony count, survival rate and results of cultures of the blood, spleen and liver of the non-immunized mice. The blood cultures of 8%, 32%, 32% and 12% of the non-immunized mice were positive after 2, 3, 4 and 6 days post-inoculation, respectively. The spleen and liver cultures were positive in 76% of the mice who died within 6 days of inoculation. Exotoxin A was detected in their sera 2 days post-infection and remained detectable for 6 days.

**Table 2:** Survival rates, presence of exotoxin A, culture results and colony counts in the control group (non-immunized mice) inoculated with *P. aeruginosa*

Post-inoculation time (day)	Number of animals alive (survival rate, %)	CFU/mL from inoculated burns	Exotoxin A in sera (%)*	Positive culture (%)		
				Liver	Spleen	Blood
1	25 (100)	1 × 10^8^	-	-	-	-
2	25 (100)	1.14 × 10^8^	2 (8)	-	-	2 (8)
3	12 (48)	1.25 × 10^8^	8 (32)	2 (8)	2 (8)	8 (32)
4	8 (32)	1.6 × 10^8^	8 (32)	8 (32)	8 (32)	8 (32)
6	5 (20)	1.7 × 10^8^	3 (12)	5 (20)	5 (20)	3 (12)

* detected with CIEP

Table 3 shows the colony count, survival rate, quantity of exotoxin and anti-exotoxin A and the result of cultures of the blood, spleen and liver of the mice in the experimental group. As expected, no exotoxin A was detected in the sera by CIEP, which may be due to neutralization of the toxin by previously antitoxins formed following immunization. Bacterial infection is a major complication after thermal injury, especially in developing countries [16-18]. 75% of deaths following burns are related to microbial infections [19]. *P. aeruginosa *is a frequently isolated bacterium that causes septicemia and death [17]. It is a ubiquitous opportunistic, non-fermenting, gram-negative rod that can infect patients with impaired immune systems. Treatment of *P. aeruginosa *infection is frequently hindered by antibiotic resistance, and multi-drug resistant strains are mostly isolated from burn wound infections [3,4,20]. An efficient vaccine is therefore needed. After colonizing the site of the burn, *P. aeruginosa *produces several virulence factors, such as exotoxin A, alkaline protease and elastase, which affect the host tissue. High titers of antitoxin against exotoxin A in patients infected with *P. aeruginosa *reduces the risk septicemia and death [9,21].

**Table 3:** Survival rates, presence of exotoxin A, culture results and colony counts in the experimental group (immunized mice) inoculated with *P. aeruginosa*

Post-inoculation time (day)	Number of animals alive (survival rate, %)	CFU/mL from inoculated burns	Exotoxin A in sera (%)*	Positive culture (%)	Number of animals alive (survival rate, %)		
					Liver	Spleen	Blood
1	48 (100)	1.5 × 10^8^	ND	48 (100)	-	-	-
4	48 (100)	1.4 × 10^7^	ND	48 (100)	-	-	-
7	47 (98)	1.3 × 10^6^	ND	47 (100)	1 (2)	1 (2)	1 (2)
11	46 (96)	1.2 × 10^5^	ND	47 (98)	1 (2)	1 (2)	1 (2)
14	45 (94)	1 × 10^4^	ND	45 (94)	1 (2)	1 (2)	1 (2)

ND, not detectable by CIEP; * neutralizing antibody detected

## Conclusion

Exotoxin A is the principal lethal factor of *P. aeruginosa*. It seems logical that a toxoid of exotoxin A could be used as an effective vaccine. Our study shows that in mice immunized with semi-purified exotoxin A, a protective titer of antitoxin developed that effectively prevented the experimentally infected animals from septicemia and death. The majority (93.8%) of immunized infected mice survived during 70 days of observation after a burn wound was inoculated with *P. aeruginosa *while all the non-immunized mice in the control group died. The rising antibody titer in the surviving mice and the decrease in the mortality rate indicate the presence of an effective antitoxin in the immunized mice.

Pavlovskis et al. [22] found that the survival rate did not increase significantly following active immunization with a toxoid of exotoxin A and infection with *P. aeruginosa *in burned mice. However, Matsumato et al. [5] found that immunization with a combination of alkaline protease and toxoid of exotoxin A decreased mortality. Some investigators have reported that active immunization with a lipopolysaccharide and an outer membrane protein (OMP) of *P. aeruginosa *could control the infection in the burned area [23,24]. Our study, using a semi-purified exotoxin A that contained trace amounts of LPS and OMP, points to a higher efficacy than a toxoid prepared from purified exotoxin A. Our study did not include different strains of the bacterium but our results can be used for further studies on the purification of, and determination of cross-immunization of, different strains of the bacterium [25].

Three of the immunized mice (6.3%) died. This may be due to the presence of invasive factors other than exotoxin A, such as elastase, alkaline protease, hemolysins, leukocidin, siderophores, siderophore uptake systems and pyocyanin diffusible pigment. Passive immunization was not evaluated in this study: We chose to study active immunization because this could play a role in high-risk occupations such as fire fighting and baking. Our results demonstrate that in a mouse model of bacterial infection in burn wounds, active immunization with semipurified exotoxin A protected against infection with *P. aeruginosa *and reduced mortality.

## Authors' contributions

AM plastic surgeon, main researcher, cooperated in inducing burns. JK microbiologist, immunological methods. DM laboratory animal design, manuscript draft provision. AJ microbiologist, bacteriological methods. MA general surgeon, cooperated in inducing burns. MN assistant in bacteriological methods. AHZ assistant surgeon and laboratory animal carer. NK assistant in immunological methods
